# Balancing Bleeding and Thrombosis: A Rare Case of Hereditary Hemorrhagic Telangiectasia With Antiphospholipid Syndrome

**DOI:** 10.7759/cureus.97182

**Published:** 2025-11-18

**Authors:** Revathi K, Pruthvi J, Ranganatha R, Murali Mohan B.V, Syed Tousheed

**Affiliations:** 1 Internal Medicine, Narayana Health, Bengaluru, IND; 2 Internal Medicine, Sri Venkateswara Medical College, Tirupati, IND; 3 Pulmonology, Narayana Health, Bengaluru, IND; 4 Pulmonary Medicine, Narayana Health, Bengaluru, IND

**Keywords:** anticoagulation, antiphospholipid syndrome, hereditary hemorrhagic telangiectasia, membranous nephropathy, pulmonary avm, sjogren’s syndrome

## Abstract

Hereditary hemorrhagic telangiectasia (HHT) is a rare autosomal dominant vascular disorder characterized by recurrent epistaxis, mucocutaneous telangiectasias, and visceral arteriovenous malformations (AVMs). Antiphospholipid syndrome (APS) is an autoimmune prothrombotic condition that necessitates long-term anticoagulation. The coexistence of these two entities presents a unique therapeutic paradox, as treatment of one often worsens the complications of the other. We report the case of a 44-year-old man with recurrent childhood-onset epistaxis, mucocutaneous telangiectasias, and a pulmonary AVM, with a positive family history fulfilling the Curaçao criteria for HHT. He presented with breathlessness, and imaging revealed necrotising pneumonia, pulmonary AVM, renal vein thrombosis, and chronic ischemic stroke. Autoimmune testing confirmed secondary APS associated with Sjögren’s disease. The coexistence of active necrotizing pneumonia and AVM made the initiation of anticoagulation unsafe due to the high bleeding risk. Following clinical stabilization and successful AVM embolisation, anticoagulation with a vitamin K antagonist was cautiously initiated under close multidisciplinary supervision. One year later, the patient developed PLA2R-positive membranous nephropathy, which was managed with rituximab. This rare coexistence of HHT and APS underscores the challenge of balancing thrombotic prevention against bleeding risk. Definitive AVM management, individualized anticoagulation, and vigilant long-term multidisciplinary follow-up are essential to achieving optimal outcomes in such complex cases.

## Introduction

Hereditary hemorrhagic telangiectasia (HHT), or Osler-Weber-Rendu syndrome, is a rare autosomal dominant vascular disorder with a prevalence of approximately one in 5,000-8,000 individuals [1]. It is characterized by recurrent epistaxis, mucocutaneous telangiectasias, and visceral arteriovenous malformations (AVMs), particularly in the lungs, liver, and brain [1]. The disease carries a high morbidity burden due to complications such as chronic anemia, paradoxical emboli, hypoxemia, and life-threatening hemorrhage. Diagnosis relies on the Curacao criteria [1]. They include four key features: recurrent spontaneous epistaxis, mucocutaneous telangiectasias, AVMs, and a first-degree relative with HHT. A diagnosis is considered definite when three or more criteria are present, possible with two, and unlikely with fewer than two.

Antiphospholipid syndrome (APS) is an acquired autoimmune thrombophilia defined by venous or arterial thrombosis, recurrent pregnancy morbidity, and persistent positivity of antiphospholipid antibodies [2]. It may occur as a primary entity or secondary to systemic autoimmune diseases such as lupus and Sjogren’s syndrome [2]. Long-term anticoagulation, usually with vitamin K antagonists, is the standard of care. For venous events, an international normalised ratio (INR) of 2.0-3.0 is recommended, while arterial events or recurrences may necessitate higher-intensity anticoagulation (INR 3.0-4.0) or combination with low-dose aspirin [2-5].

The coexistence of HHT and APS is exceedingly rare, with only isolated cases documented [3]. This dual pathology creates a therapeutic paradox: APS mandates lifelong anticoagulation to prevent thrombosis, whereas HHT predisposes to recurrent bleeding, particularly from epistaxis and gastrointestinal telangiectasias [1,2]. Reports and reviews indicate that antithrombotic therapy can be used in HHT but often worsens bleeding manifestations [1,6,7], and observational data confirm higher hospitalization rates for bleeding in anticoagulated HHT patients [7]. Direct oral anticoagulants (DOACs) have been trialed with some success in HHT [6], but in APS, especially high-risk or triple-positive cases, vitamin K antagonists remain the standard [4].

Here, we describe a rare case of HHT with secondary APS and Sjogren’s disease, further complicated by membranous nephropathy. The case emphasizes the challenge of anticoagulation in patients with simultaneous bleeding and thrombotic risks and highlights the importance of multidisciplinary, individualized management.

## Case presentation

A 44-year-old male presented with fever and exertional dyspnea. Evaluation revealed necrotizing pneumonia, pulmonary AVM, and renal vein thrombosis in the background of recurrent epistaxis since childhood occurring five to 10 times per month and mucocutaneous telangiectasias suggestive of hereditary hemorrhagic telangiectasia. His family history was significant for similar complaints in his mother. Past history included hypertension, COVID-19 pneumonia, an old ischemic stroke detected on CT brain, and migraine.

**Figure 1:**
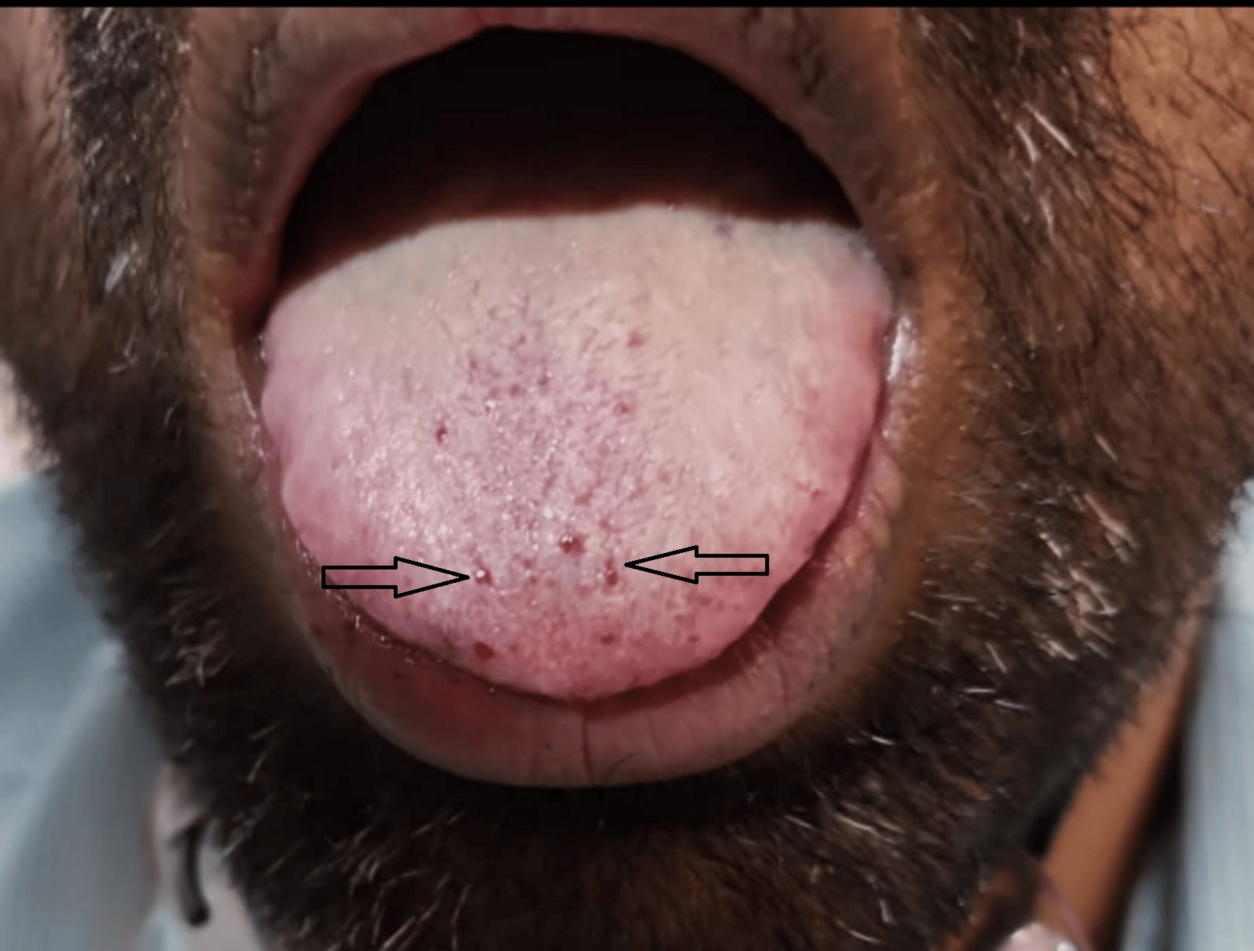
Tongue showing multiple mucocutaneous telangiectasias characteristic of hereditary hemorrhagic telangiectasia (HHT)

**Figure 2:**
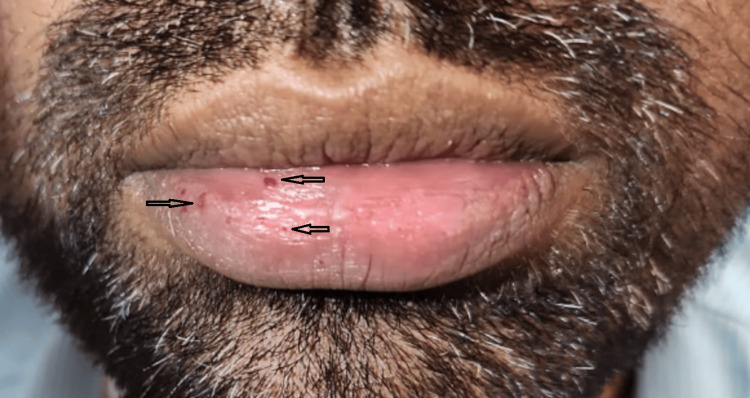
Lips showing multiple mucocutaneous telangiectasias characteristic of hereditary hemorrhagic telangiectasia (HHT)

On admission, he was afebrile with a blood pressure of 140/80 mmHg, heart rate 78/min, respiratory rate 18/min, and oxygen saturation of 88% on room air. He had active epistaxis and multiple mucocutaneous telangiectasias on the lips and tongue (Figures 1, 2). Cardiovascular examination revealed a grade 3/6 pansystolic murmur, and respiratory examination revealed bilateral basal crepitations.

Baseline investigations (Table 1) showed hemoglobin 15 g/dL, WBC count: 7.4 × 10⁹/L with markedly elevated inflammatory markers C-reactive protein (CRP; 178).

**Table 1:** Baseline investigation

Parameter	Patient Value	Reference Range	Remarks
Hemoglobin	15 g/dL	12–16 g/dL (female), 13–17 g/dL (male)	Within normal limits
Total Leukocyte Count	7.4 × 10⁹/L	4.0–11.0 × 10⁹/L	Within normal limits
C-Reactive Protein (CRP)	178 mg/L	<5 mg/L	Markedly elevated
Platelet Count	350 × 10⁹/L	150–450 × 10⁹/L	Within normal limits
Serum Creatinine	0.8 mg/dL	0.6–1.2 mg/dL	Within normal limits
Random Blood Glucose	105 mg/dL	70–140 mg/dL	Within normal limits
Liver Function Tests	—	Within normal limits	—

**Figure 3:**
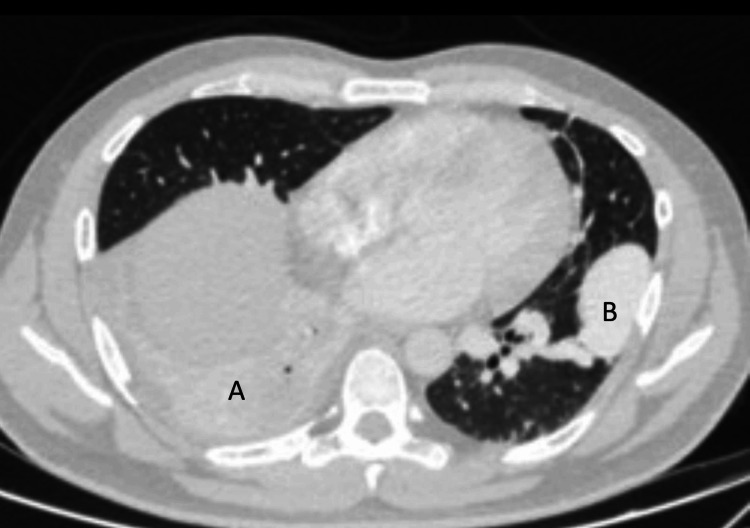
CT thorax plain (lung window) A- Collapse consolidative changes with air bronchogram and focal cavitatory lesion (3.2 x 2.4 cm) in right lower lobe, suggestive of infective etiology. B- Homogenously enhancing, well circumscribed, juxtafissural soft tissue density nodule in left lung with feeding artery as segmental branch from left lower lobar artery and draining vein as left inferior pulmonary vein. Features are suggestive of pulmonary arteriovenous malformation with nidus.

**Figure 4:**
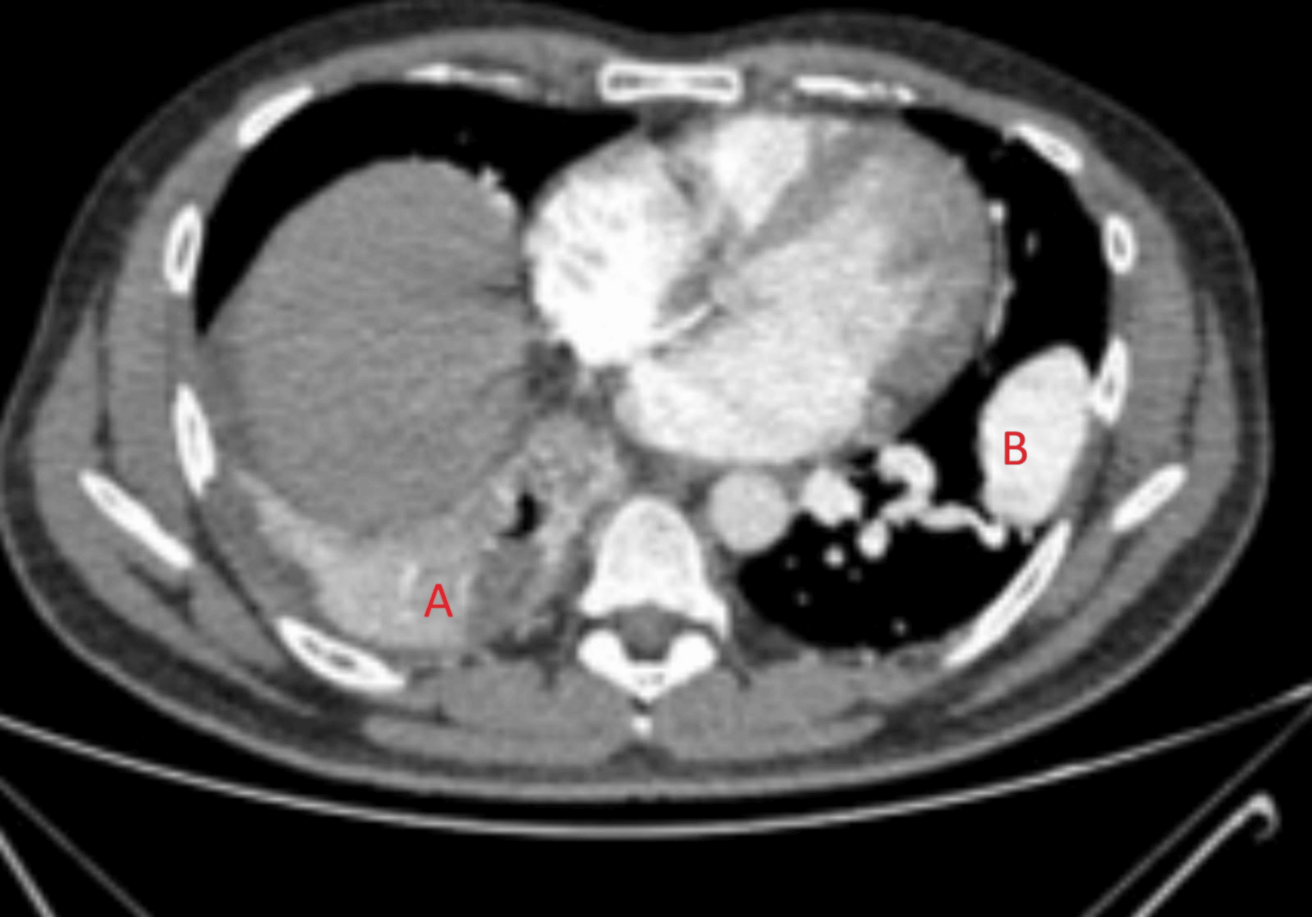
CT thorax with contrast (mediastinal window) A- Collapse consolidative changes with air bronchogram and focal cavitatory lesion (3.2 x 2.4 cm) in right lower lobe, suggestive of infective etiology. B- Homogenously enhancing, well circumscribed, juxtafissural soft tissue density nodule in left lung with feeding artery as segmental branch from left lower lobar artery and draining vein as left inferior pulmonary vein. Features are suggestive of pulmonary arteriovenous malformation with nidus.

**Figure 5:**
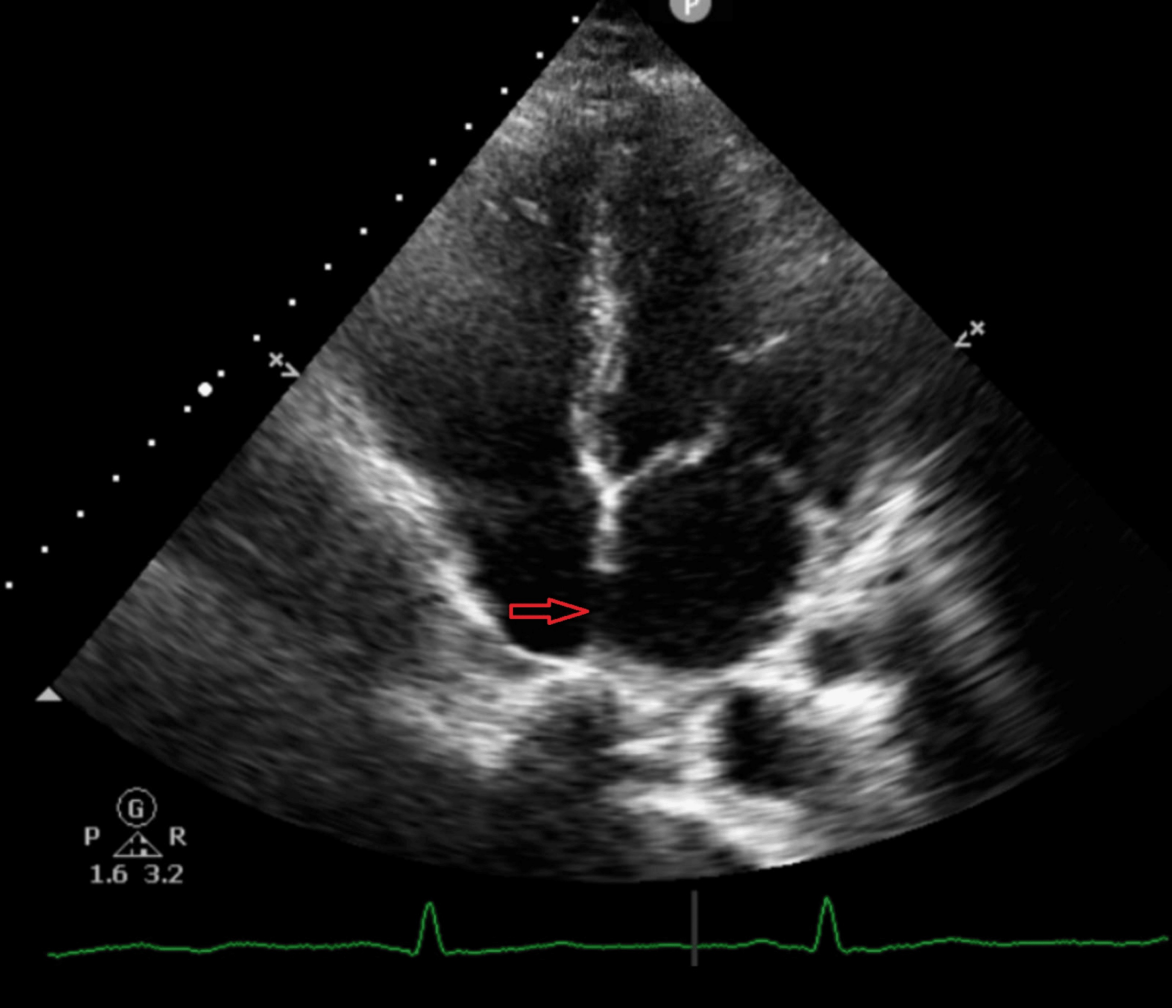
Echocardiogram (four chamber view) Transthoracic echocardiogram showing a patent foramen ovale in the interatrial septum.

Imaging studies demonstrated a homogenously enhancing, well-circumscribed juxta-fissural soft-tissue density nodule in the left lung supplied by a segmental branch of the left lower lobar artery and draining into the left inferior pulmonary vein, consistent with a pulmonary AVM with nidus. Additional findings included collapse-consolidation with air bronchograms and a cavitary lesion (3.2 x 2.4 cm) in the right lower lobe, suggestive of necrotizing pneumonia (Figures 3, 4). Abdominal imaging revealed chronic left renal vein thrombosis extending to the intrahepatic inferior vena cava, mild hepatomegaly, and small hypodense cortical lesions in the left kidney. Echocardiography with bubble contrast demonstrated a patent foramen ovale (2 mm) with significant right-to-left shunting (Figure 5).

During hospitalization, he was treated with broad-spectrum intravenous antibiotics (piperacillin-tazobactam), doxycycline, nebulization, antihypertensives, and supplemental oxygen. Epistaxis was managed conservatively by the otolaryngology team. Interventional radiology and thoracic surgery deferred AVM embolization until infection resolution. Cardiology advised postponing closure of the patent foramen ovale until neurologic evaluation. The patient improved symptomatically and was discharged on home oxygen with close follow-up.

Subsequent neurological evaluation for chronic small-vessel ischemic infarcts in this young male revealed no evidence of cerebral or dural AVMs on MRI brain. This prompted an autoimmune workup, which demonstrated coarse granular antinuclear antibody (ANA) positivity with strong anti-SSA/Ro and anti-SSB/La antibodies, along with persistent β₂-glycoprotein IgM and IgG positivity on two occasions, 12 weeks apart, while lupus anticoagulant and anticardiolipin antibodies were negative. In the absence of sicca symptoms or salivary gland biopsy, these findings were consistent with secondary Sjogren’s disease associated with APS. Given the paradox of thrombotic risk in APS and bleeding tendency in HHT, anticoagulation posed a significant therapeutic dilemma. A multidisciplinary team (hematology, rheumatology, cardiology, and interventional radiology) carefully weighed risks and benefits, and it was decided to first proceed with pulmonary AVM embolization to reduce bleeding potential. Only after successful embolization was systemic anticoagulation cautiously initiated, alongside hydroxychloroquine, with close monitoring.

On follow-up one year later, the patient developed nephrotic-range proteinuria. Renal biopsy confirmed PLA2R-positive membranous nephropathy. Rituximab therapy was initiated, with good tolerance and no bleeding episodes during follow-up. He continues to remain under vigilant multidisciplinary follow-up.

## Discussion

HHT is primarily a hemorrhagic condition, while APS is strongly prothrombotic, and their coexistence is rarely reported in the literature, with one case describing HHT with APS and factor V Leiden mutation [3]. Our patient fulfilled the diagnostic criteria for HHT with recurrent childhood-onset epistaxis, mucocutaneous telangiectasias, pulmonary AVM, and a family history of similar complaints. The presence of renal vein thrombosis, ischemic stroke, and positive antiphospholipid antibody profile confirmed APS, while anti-Ro/La positivity suggested underlying Sjogren’s disease. This multisystem overlap highlights the unique complexity of the case.

To our knowledge, very few cases of HHT coexisting with APS have been described in the literature. Table 1 summarizes the only previously published case alongside our report.

**Table 2:** Reported Cases of Hereditary Hemorrhagic Telangiectasia (HHT) Associated with Antiphospholipid Syndrome (APS) VKA: Vitamin K antagonists

Author / Year	Patient Characteristics	HHT Features	APS / Thrombotic Events	Treatment	Outcome
Undas A, et al., 2002 [3]	57-year-old woman	Clinical HHT; family history positive	Multiple ischemic strokes; anticardiolipin IgM +; anti-prothrombin IgG +; Factor V Leiden mutation	Ticlopidine (antiplatelet) instead of warfarin due to severe epistaxis	No recurrent ischemic events at 18-month follow-up
Present Case, 2023–2024	44-year-old man	Recurrent epistaxis since childhood, mucocutaneous telangiectasias, pulmonary AVM	Renal vein thrombosis, ischemic stroke; strong anti-Ro/La and β2-glycoprotein IgM/IgG positivity (secondary APS with Sjögren’s)	Hydroxychloroquine, anticoagulation (VKA), AVM embolization, rituximab for membranous nephropathy	Stable on follow-up with no major bleeding or thrombotic recurrence

The management dilemma centred on anticoagulation. The patient presented with necrotizing pneumonia, pulmonary AVM, renal vein thrombosis, and prior ischemic stroke, illustrating a high thrombotic burden. However, recurrent epistaxis and pulmonary AVM carried a significant bleeding risk. Initial treatment prioritized infection control and supportive care, with AVM embolization deferred until stabilization. Embolization subsequently reduced hemorrhagic risk to some extent, allowing us to initiate anticoagulation with lesser risk of bleeding, alongside hydroxychloroquine, which offers immunomodulatory and antithrombotic benefits.

HHT patients have dual vulnerabilities: fragile telangiectasias and AVMs predisposing to bleeding, and paradoxical emboli creating thrombotic risk [4]. Reviews and systematic analyses confirm that anticoagulation in HHT often exacerbates bleeding [1,6]. A nationwide study also demonstrated higher rates of hospitalization for bleeding among HHT patients receiving antiplatelet or anticoagulant therapy [7]. DOACs have shown tolerability in some HHT cohorts [8], but for APS, particularly with arterial events or triple antibody positivity, current recommendations discourage their use in favor of vitamin K antagonists [2]. When anticoagulation is essential, experts recommend maintaining INR at the lower therapeutic limit (≈2.0-2.5) to minimize bleeding risk [1,5]. In our patient, careful INR titration and multidisciplinary follow-up allowed effective thrombosis prevention without catastrophic bleeding.

The later development of PLA2R-positive membranous nephropathy requiring rituximab illustrates the evolving nature of autoimmune and vascular overlap syndromes. Such cases demand long-term surveillance and adaptation of therapy as new organ involvement arises.

In summary, this case underscores the paradox of managing concurrent thrombotic and hemorrhagic risks in HHT with APS. Definitive AVM management, tailored anticoagulation, and coordinated multidisciplinary care are essential for optimizing outcomes in such rare and complex presentations.

## Conclusions

We describe a rare case of HHT complicated by APS, Sjogren’s disease, and membranous nephropathy, highlighting the paradoxical coexistence of bleeding and thrombotic risks. This case underscores the therapeutic dilemma of initiating anticoagulation in patients predisposed to hemorrhage and the importance of a carefully individualized approach. Definitive management of AVMs prior to anticoagulation, combined with close hematologic, rheumatologic, and interventional follow-up, allowed safe and effective therapy. The case further emphasizes the need for ongoing multidisciplinary collaboration, patient education regarding bleeding symptoms, and regular surveillance for evolving autoimmune or vascular complications. Early recognition, risk stratification, and tailored treatment strategies are essential to improve long-term outcomes in such complex, overlapping systemic disorders.
